# Left bundle branch area pacing in mildly reduced heart failure: A systematic literature review and meta‐analysis

**DOI:** 10.1002/clc.24028

**Published:** 2023-05-05

**Authors:** Ga‐In Yu, Tae‐Hoon Kim, Yun‐Ho Cho, Jae‐Seok Bae, Jong‐Hwa Ahn, Jeong Yoon Jang, Yong Whi Park, Choong Hwan Kwak

**Affiliations:** ^1^ Division of Cardiology, Department of Internal Medicine, GyeongSang National University Changwon Hospital Gyeongsang National University College of Medicine Changwon Republic of Korea; ^2^ Division of Cardiology, Department of Internal Medicine, Severance Hospital Yonsei University College of Medicine Seoul Republic of Korea

**Keywords:** cardiac resynchronization therapy, heart failure with mildly reduced ejection fraction, left bundle branch area pacing, left ventricular ejection fraction, meta‐analysis, QRS duration

## Abstract

Cardiac resynchronization therapy (CRT) strategy for heart failure with mildly reduced ejection fraction (HFmrEF) is controversial. Left bundle branch area pacing (LBBAP) is an emerging pacing modality and an alternative option to CRT. This analysis aimed to perform a systematic review of the literature and meta‐analysis on the impact of the LBBAP strategy in HFmrEF, with left ventricular ejection fraction (LVEF) between 35% and 50%. PubMed, Embase, and Cochrane Library were searched for full‐text articles on LBBAP from inception to July 17, 2022. The outcomes of interest were QRS duration and LVEF at baseline and follow‐up in mid‐range heart failure. Data were extracted and summarized. A random‐effect model incorporating the potential heterogeneity was used to synthesize the results. Out of 1065 articles, 8 met the inclusion criteria for 211 mid‐range heart failure patients with an implant LBBAP across the 16 centers. The average implant success rate with lumenless pacing lead use was 91.3%, and 19 complications were reported among all 211 enrolled patients. During the average follow‐up of 9.1 months, the average LVEF was 39.8% at baseline and 50.5% at follow‐up (MD: 10.90%, 95% CI: 6.56−15.23, *p* < .01). Average QRS duration was 152.6 ms at baseline and 119.3 ms at follow‐up (MD: −34.51 ms, 95% CI: −60.00 to −9.02, *p* < .01). LBBAP could significantly reduce QRS duration and improve systolic function in a patient with LVEF between 35% and 50%. Application of LBBAP as a CRT strategy for HFmrEF may be a viable option.

## Graphical abstract

Average left ventricular ejection fraction (LVEF) from implant to follow‐up for baseline LVEF between 35% and 50% versus LVEF <35%.

▪ Left bundle branch area pacing (LBBAP) was found to significantly shorten QRS duration and improve systolic cardiac function in patients with a LVEF of 35%−50%.

▪ In patients with heart failure with mildly reduced ejection fraction, LBBAP resulted in a significant increase in ejection fraction.

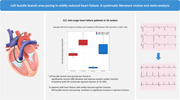

## 1 INTRODUCTION

The advantageous effect of cardiac resynchronization therapy (CRT) is well known in heart failure (HF) patients with reduced left ventricular ejection fraction (LVEF) and prolonged QRS duration.^1^, ^2^, ^3^ Current global guidelines suggest CRT for class I or IIA indications in patients with LVEF ≤35% and symptomatic HF despite receiving optimal drug therapy.^4^, ^5^, ^6^ However, an LVEF cut‐off of ≤35% was determined by the patient enrollment criteria in major CRT trials, and of note, was adopted from cut‐off values in prior major implantable cardiac defibrillator trials rather than from a prospective risk‐benefit analysis of CRT for all LVEF ranges.

Although resynchronization therapy for HF patients with LVEF ≥35% is controversial and has not been clearly established, HF with LVEF >35% shows disease features similar to LVEF <35%, and treatment patterns are similar.^7^, ^8^ Additionally, there are reports that long‐term clinical outcomes were poor when HF with mildly reduced ejection fraction (HFmrEF) was accompanied by left bundle branch block (LBBB) versus without LBBB.^9^ Moreover, in a retrospective analysis of the PROSPECT trial database, CRT demonstrated significant clinical benefit among patients with an LVEF >35%.^10^ The BLOCK‐HF trial proved that CRT provided a clinical benefit over right ventricular pacing in patients with LVEF ≤50% and atrioventricular block who require ventricular pacing, and almost 70% (483/691) of the study population had an LVEF of 36%−50%.^11^

Conduction system pacing (CSP) that directly activates the specialized conduction system was developed, and left bundle branch area pacing (LBBAP)—which overcomes the limitations of the previously used His bundle pacing (HBP)—is emerging and widely used.^12^, ^13^, ^14^, ^15^, ^16^

Furthermore, the effectiveness and safety of LBBAP in patients with HF have also been reported.^17^, ^18^, ^19^ To date, LBBAP has been used as an alternative to CRT in indicated patients, as well as a first option for patients indicated for CRT or pacemaker implantation.^17^, ^18^, ^19^ LBBAP is more simple, convenient and cheaper than biventricular CRT; therefore, there is increasing clinical interest in adopting wider LVEF ranges for LBBAP‐CRT among patients with HF and a long QRS duration, especially in patients with an LVEF of 36%−50%. Therefore, we aimed to systematically review the literature and meta‐analysis on the impact of the LBBAP strategy in patients with HFmrEF with an LVEF between 35% and 50%.

## 2 METHODS

### 2.1 Data sources and searches

This systematic review and meta‐analysis were carried out following the Preferred Reporting Items for Systematic Reviews and Meta‐Analyses (PRISMA) statement.^20^ Searches were independently performed by two investigators (G. Y. and T. K.) who searched Embase, Medline (PubMed), and Cochrane's Library databases for related articles, with the keywords: “left bundle branch pacing” OR “left bundle branch area pacing.” At the same time, references of the relevant original and review articles were manually sorted to undergo a comprehensive search. The search was restricted to research in humans published in English, and was completed on July 17, 2022.

### 2.2 Study selection and data extraction

Titles and abstracts were retrieved from articles searched using the keywords “left bundle branch pacing” or “left bundle branch area pacing.” Studies were included if the article was in English and met the following criteria: patients aged >18 years; included patients with an LVEF from 35% to 50%; LVEF or QRS duration follow‐up was performed after the procedure; and for LBBAP CRT, both patients with pacing indications (de novo or upgrade), and with HF without pacing indications (de novo CRT), were included. Among studies satisfying the above criteria, a pooled analysis of patients with a baseline LVEF of 35%−50% was used. Editorials, review articles, case reports/letters, and abstracts were excluded. The quality of the literature was evaluated by the above two researchers using the Newcastle−Ottawa scale criteria.^21^

### 2.3 Statistical analyses

To evaluate the change in LVEF and QRS duration over time, means, standard deviations, and sample sizes were extracted from articles to estimate the overall average values and confidence intervals (CIs). Statistical heterogeneity was conducted by calculating Higgins *I* ^2^; *I* ^2^ > 50% was considered to indicate significant heterogeneity.^22^ After confirming heterogeneity, we used a random effects model because the populations included in the individual studies were from different populations.^23^ Potential publication bias was assessed by visual inspection of the funnel plot and the Egger regression asymmetry test. If the *p* for bias was >.05, it was judged that there was no publication bias.^24^ All tests with *p* < .05 were considered to be statistically significant. Statistical analyses were performed using R programming version 4.0.3 (The R Foundation for Statistical Computing).

## 3 RESULTS

### 3.1 Studies selection and evaluation

**Figure 1:**
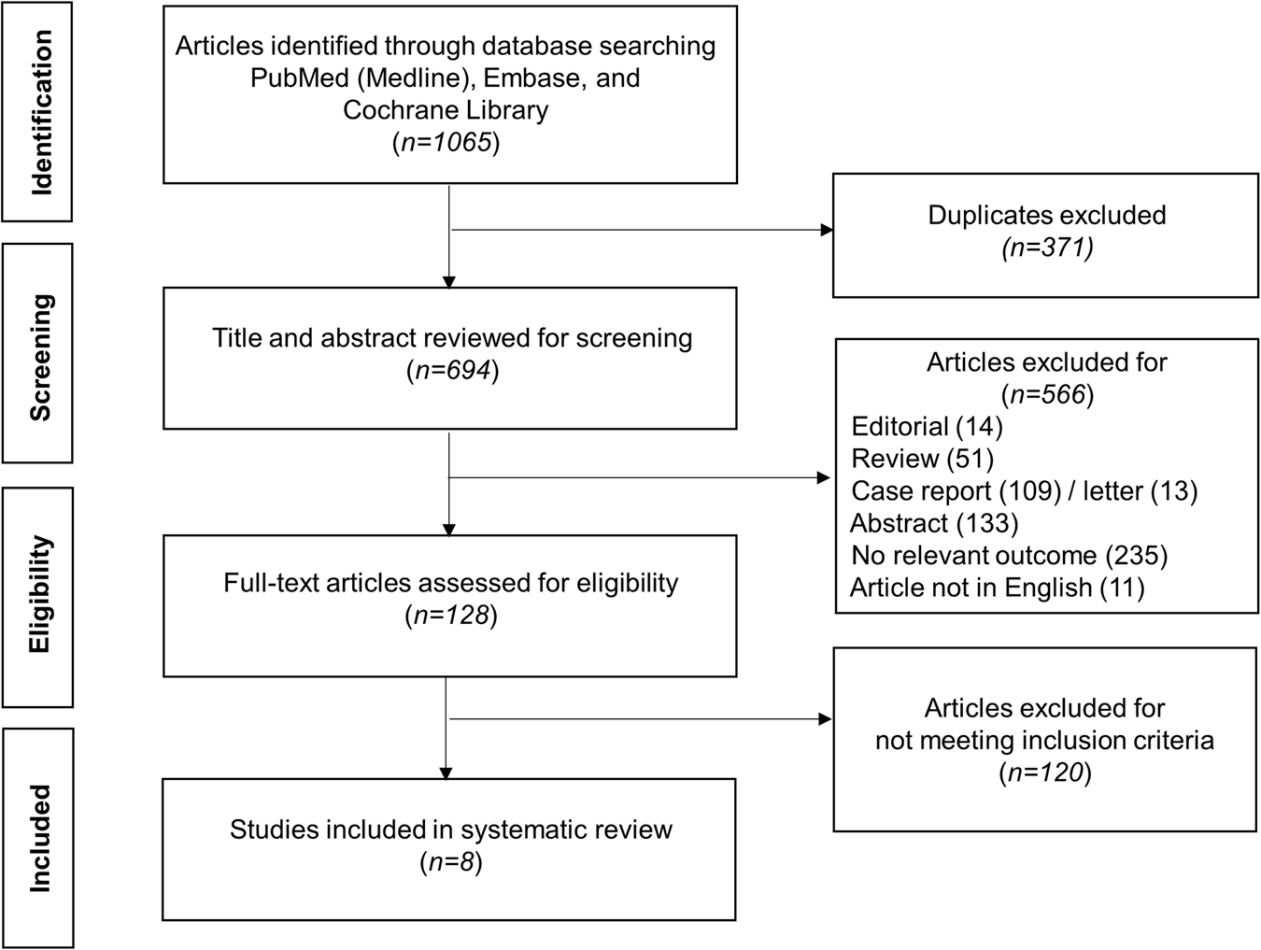
Flow diagram of selection process for articles included in the meta‐analysis.

In total, 694 articles were retrieved after excluding duplicates. The title and abstract of the articles were screened, and 566 were excluded due to being editorials/review articles, case reports/letters, having no relevant outcome, and not being in English. The remaining 128 articles underwent full‐text review, and 120 were excluded for not meeting the inclusion criteria; finally, eight studies^25^, ^26^, ^27^, ^28^, ^29^, ^30^, ^31^, ^32^ were retrieved (Figure 1 and Table 1).

**Table 1:** Basic characteristics of the included studies.

Author	Year	Article type	Study design	Indication	Pacing lead	Implant success (%)	Number of Pt with HFmrEF (*n*)	Male (%)	Age (years)	Follow‐up (months)
Li et al.^25^	2020	Original article	Retrospective observational/comparative (LBBB vs. RBBB)	Bradycardia with BBB	LLL^a^	83.6	16	67.3	60.9 ± 10.9	6.0
Li et al.^26^	2020	Original article	Prospective cohort/single‐arm	HF	LLL	100	4	25.0	62.3 ± 17.3	9.3
Qian et al.^27^	2020	Original article	Retrospective observational/comparative (HFmrEF vs. HFpEF)	PICM	LLL	93.3	13	69.2	75.8 ± 6.8	10.4
Ponnusamy et al.^28^	2021	Original article	Retrospective observational/single‐arm	LBBB‐induced CMP	LLL	NR^b^	4	25.0	59.8 ± 17.5	12.7
Wei et al.^29^	2021	Original article	Retrospective observational/single‐arm	Conduction disorder following valve op	LLL	90.9	4	100.0	57.5 ± 4.8	16.1
Vijayaraman et al.^30^	2021	Original article	Retrospective observational/single‐arm	HF with LBBB	LLL	85.0	146	64.0	70.0 ± 13.0	6.0
Jiang et al.^31^	2022	Original article	Retrospective observational/comparative (HFmrEF vs. HFrEF)	HF with LBBB	LLL	77.8	17	42.9	70.0 ± 8.0	6.0
Rademakers et al.^32^	2022	Original article	Retrospective observational/single‐arm	PICM	LLL	100	7	70.0	77.0 ± 10.0	6.0

*Note*: Values are presented as mean ± standard deviation, or *n* (%).

Abbreviations: BBB, bundle branch block; HF, heart failure; HFmrEF, heart failure with mid‐range ejection fraction; LBBB, left bundle branch block; PICM, pacing‐induced cardiomyopathy; RBBB, right bundle branch block.

a SelectSecure pacing lead (Model 3830, 69 cm, Medtronic Inc.)

^b^ Only included successful implants.

The eight articles included five single‐arm studies, and three comparative studies, published between 2020 and 2022. After pooled analysis, the total population included 211 patients with HF and an LVEF of 35%−50%, who underwent LBBAP at 1 of 16 centers in seven countries. The average age was 65.1 years (*n* = 211; 95% CI: 58.4−71.8), and 52.4% (*n* = 211; 95% CI: 24.2−80.6) of patients were male. The average baseline QRS duration and LVEF were 153 ms (*n* = 2; 95% CI: 133−172) and 40% (*n* = 207; 95% CI: 38−41), respectively. The average follow‐up period was 9.1 ± 3.8 months. Regarding quality assessment, the Newcastle−Ottawa scale criteria were used to evaluate the quality of observational studies (Supporting Information: Table S1).

### 3.2 Definition of LBBAP

The definition of LBBAP was described in seven studies and omitted in one. Confirmation of LBBAP was made by comprehensively reviewing the following findings: (1) the only criterion used in all seven studies was paced QRS morphology, presented with a right bundle branch block morphology pattern in lead V1; (2) abrupt shortening of Stim‐LVAT (stimulus to a peak of the R wave) in leads V5 and V6 was used in four studies; (3) observed left bundle branch potential in pacing leads was used in four studies; and (4) demonstration of the transition from nonselective LBBP to selective LBBP was used in two studies. Only one study used contrast to ensure the location of the lead in the interventricular septum.

### 3.3 Changes in QRS duration and left ventricular systolic function

**Figure 2:**
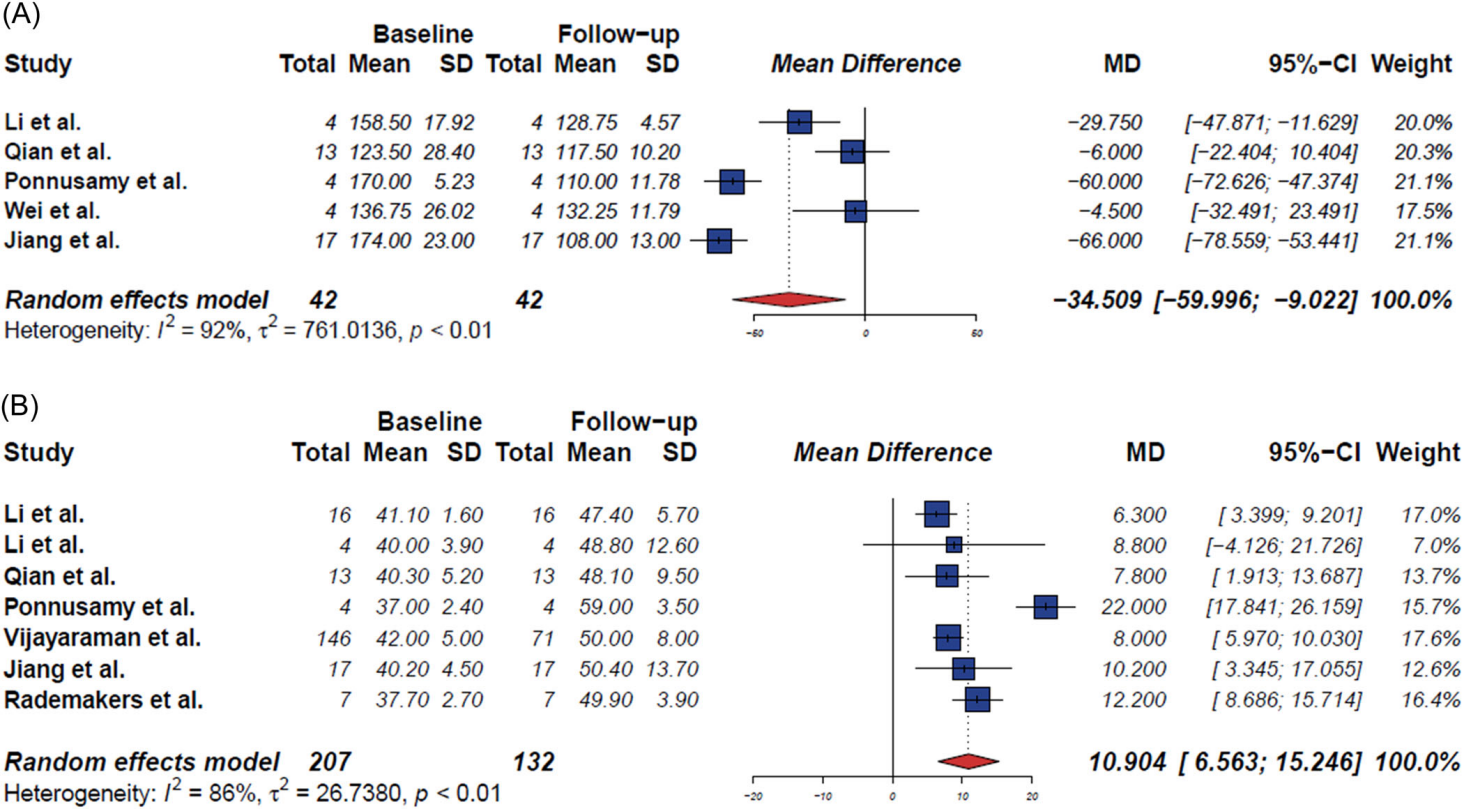
Forest plot for the meta‐analysis comparing impact of LBBAP on QRS duration and LVEF. (A) Changes in QRS duration before and after LBBAP. (B) Changes in LVEF before and after LBBAP. Plot demonstrating significant reduction in QRS duration and significant improvement in LVEF. Square data markers represent mean diference of QRS duration (A) and LVEF (B) between pre implantation and postimplantation, and horizontal lines represent 95% CIs. CI, confidence interval; LBBAP, left bundle branch area pacing; LVEF, left ventricular ejection fraction; SD, standard deviation.

Five studies presented both the baseline and follow‐up QRS durations, with means and standard deviations. Pooled analysis with a random effects model showed that LBBAP was related to a significantly reduced QRS duration (MD: −34.51 ms, 95% CI: −60.00 to −9.02; *p* < .01, *I* ^2^ = 92%; Figure 2A); the duration decreased from 152.6 ± 21.8 ms (*n* = 42, 95% CI: 133.48−171.62 ms) at baseline, to 119.3 ± 10.9 ms (*n* = 42, 95% CI: 109.75−128.85 ms) after LBBAP.

**Figure 3:**
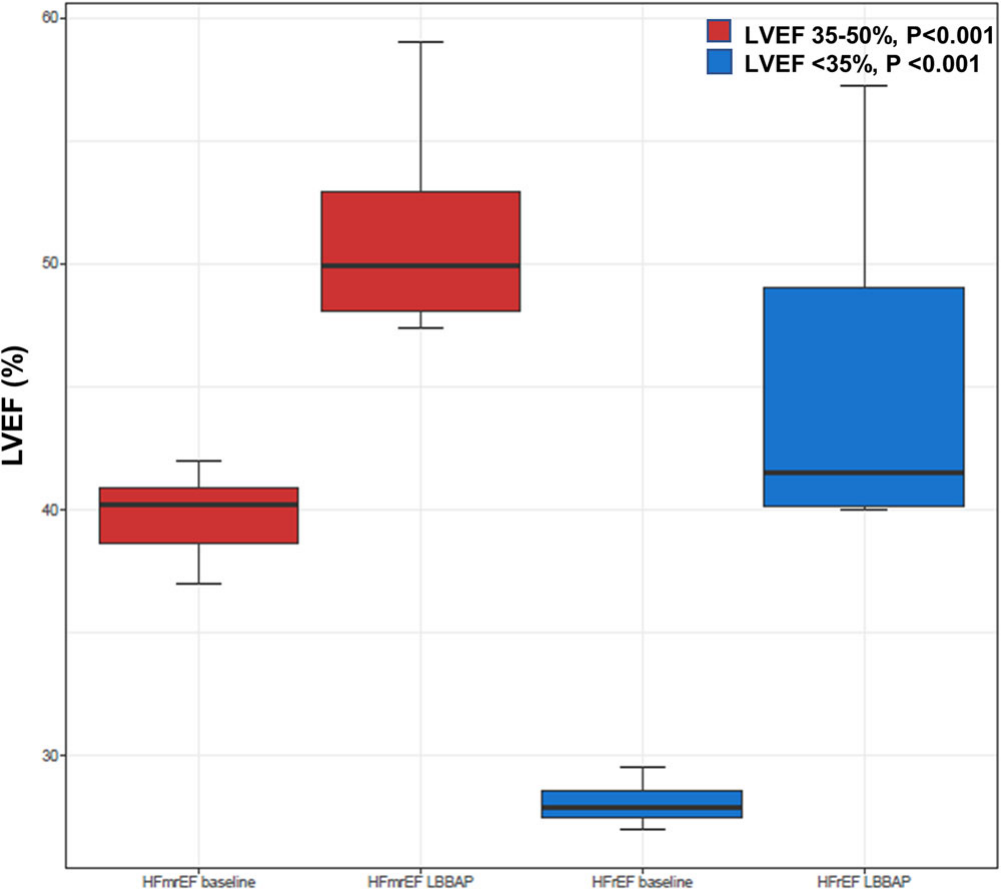
Average LVEF from implant to follow‐up for baseline LVEF between 35% and 50% versus LVEF <35%. Both HFrEF and HFmrEF exhibited significant improvements in LVEF after LBBAP. HFmrEF, heart failure with mildly reduced ejection fraction; HFrEF, heart failure with reduced ejection fraction; LBBAP, left bundle branch area pacing; LVEF, left ventricular ejection fraction.

Seven studies presented both preimplant and postimplant LVEF values, with means and standard deviations (Table 2). The average LVEF among the seven studies was 39.8 ± 1.8% at baseline (*n* = 207), and 50.5 ± 3.9% at follow‐up (*n* = 132). During the average follow‐up of 9.1 ± 3.8 months, LVEF significantly improved (MD: 10.90%, 95% CI: 6.56−15.23; *p* < .01, *I* ^2^ = 87%; Figure 2B). Five studies included patients with HF with reduced ejection fraction (HFrEF). When HFrEF and HFmrEF were compared, the estimated increase was 59.3% for HFrEF ([average LVEF] baseline, 28.1 ± 1.1%; follow‐up, 45.6 ± 7.9%) and 27.6% for HFmrEF ([average LVEF] baseline, 39.8 ± 1.8%; follow‐up, 50.5 ± 3.9%; Figure 3). Only one study compared LVEF changes in HFmrEF and HF with preserved ejection fraction; the estimated increase was 19.4% for HFmrEF (40.3 ± 5.2 to 48.1 ± 9.5; *p* = .002) and 3.9% for HF with preserved ejection fraction (59.1 ± 4.2 to 61.4 ± 4.3; *p* = .009).

**Table 2:** LVEF at baseline and follow‐up.

References	Year	Baseline		Follow‐up			Difference (mean ± SD)
		Sample size	Mean ± SD	Sample size	Follow‐up (months)	Mean ± SD	
Li et al.^25^	2020	16	41.1 ± 1.6	16	6.0	47.4 ± 5.7	6.3 ± 4.2
Li et al.^26^	2020	4	40.0 ± 3.9	4	9.3	48.8 ± 12.6	8.8 ± 9.3
Qian et al.^27^	2020	13	40.3 ± 5.2	13	10.4	48.1 ± 9.5	7.8 ± 7.7
Ponnusamy et al.^28^	2021	4	37.0 ± 2.4	4	12.7	59.0 ± 3.46	22.0 ± 4.2
Vijayaraman et al.^30^	2021	146	42.0 ± 5.0	71	6.0	50.0 ± 8.0	8.0 ± 6.1
Jiang et al.^31^	2022	17	40.2 ± 4.5	17	6.0	50.4 ± 13.7	10.2 ± 10.2
Rademakers et al.^32^	2022	7	38.8 ± 2.4	7	6.0	50.4 ± 4.3	12.2 ± 3.4
Total		207		132			
				95% CI: (1.36−36.36)	95% CI: (6.02−10.1)	95% CI: (47.71−53.47)	

*Note*: Values are presented as mean ± standard deviation, or *n* (%).

Abbreviations: CI, confidence interval; LVEF, left ventricular ejection fraction; SD, standard deviation.

### 3.4 Complications and clinical outcome

Among all studies, total complications related to the LBBAP procedure included 19 cases: 11 cases of repositioning to another location in the left bundle branch area due to septal perforation during the procedure, 3 cases of pneumothorax, 3 cases of pocket infection requiring incision and drainage, and 2 cases of pocket hematoma requiring evacuation. During the follow‐up period, 5 cases of lead dislodgements were observed.

One study reported both preimplant and postimplant New York Heart Association (NYHA) functional classes before and after LBBAP in patients with HFmrEF. In that study, during a mean follow‐up of 10.4 months, NYHA functional status in patients with HFmrEF who underwent LBBAP improved from 2.5 ± 0.5 at baseline, to 1.7 ± 0.8 at follow‐up (*p* < .01). One study reported hospitalization for HF after LBBAP in patients with HFmrEF; in this study, the HF hospitalization rate was 17.6% during a mean follow‐up of 6.0 months after LBBAP.

### 3.5 Publication bias

According to Egger regression, the *p* for bias in QRS duration and LVEF were confirmed to be 0.126 and 0.433, respectively, indicating that there was no obvious publication bias. The funnel plots of the studies included in each analysis are presented in Supporting Information: Figure S1.

## 4 DISCUSSION

The clinical importance of cardiac resynchronization in patients with HF has already been demonstrated.^1^, ^2^, ^3^ Indications for CRT in patients with HFrEF with an LVEF <35% are well established, whereas those for patients with HFmrEF and an LVEF of 35%−50% are less certain.^4^, ^5^, ^33^ In the PROSPECT prospective multicenter study, all echocardiograms were analyzed by a core laboratory; among patients with an NYHA functional Class III−IV status, and QRS >130 ms, those with a core laboratory‐measured LVEF >35% who underwent CRT demonstrated significant clinical benefit. Additionally, they exhibited both clinical and structural benefits from CRT.^34^, ^35^ Meanwhile, among patients with HF and an LVEF <50% who required pacing due to atrioventricular block, the BLOCK‐HF trial showed that CRT was superior to right ventricular pacing regarding mortality and HF hospitalization.^11^ Based on these study results, guidelines state that HBP or biventricular pacing can be considered when the ventricular pacing burden exceeds 40% in HFmrEF; still, the resynchronization strategy for HFrEF has not been clearly established.^36^ As the prevalence of HFmrEF increases, its clinical importance is emerging.^37^ Several studies have reported the feasibility, efficacy, and safety of strategies for adapting LBBAP to cardiac resynchronization using LBBAP lead instead of LV lead (LBBAP‐CRT) or both LV lead and LBBAP lead (LBBAP optimized CRT, LOT‐CRT).^17^, ^18^ However, patients with HFmrEF require further study.

### 4.1 Feasibility of LBBAP in HFmrEF

All studies included in this meta‐analysis performed LBBAP using lumenless pacing leads, with an average acute success rate of 91.3%. In 1 case series wherein LBBAP was performed in patients with HFmrEF using stylet‐driven leads, the success rate was 100% (*n* = 4).^38^ Although procedure‐related complications of septal perforation, pneumothorax, pocket infection, pocket hematoma, and lead dislodgements occurred during the follow‐up period, no major implantation‐related complications were observed.

### 4.2 LBBAP as a resynchronization strategy for HFmrEF

In this meta‐analysis, during the average follow‐up of 9.1 ± 3.8 months, the mean decrease in QRS duration after LBBAP in patients with HFmrEF was −34.51 ms (95% CI: −60.00 to −9.02; *p* < .01). The mean LVEF increase was 10.9% (95% CI: 6.56−15.23, *p* < .01), and the NYHA functional status improvement was −0.8 (only one article, *p* < .01). This suggests that LBBAP is clinically beneficial for resynchronization therapy in the treatment of HFmrEF.

Interestingly, both HFrEF and HFmrEF exhibited significant improvements in LVEF after LBBAP. The follow‐up LVEF after LBBAP was 45.6 ± 7.9% in patients with baseline HFrEF and 50.5 ± 3.9% in patients with baseline HFmrEF. This suggests that intervention before LVEF falls below 35% and before cardiac remodeling can proceed is more beneficial.

Current findings reveal that about 30% of patients for whom CRT is indicated are nonresponders.^3^, ^39^, ^40^ Moreover, among several studies that evaluated the effect of CRT in midrange HF with an LVEF >35%, CRT did not significantly increase the LVEF or clinical composite score.^34^ In the midst of this, it is encouraging that a procedure using LBBAP, which improved the limitations of CSP and HBP, showed significant improvement in LVEF in patients with HFmrEF.

### 4.3 Limitations

This analysis has several limitations. First, there are currently no randomized controlled trials for LBBAP targeting HFmrEF; therefore, all studies included in this meta‐analysis were retrospective and observational. Second, the sample size was small, with 16 centers and 211 patients (207 patients with LVEF followed‐up before and after LBBAP, 42 patients with QRS duration followed‐up before and after LBBAP) included in the analysis. Third, there was no long‐term follow‐up data. Fourth, in all studies included in the analysis, the pacing lead used for the procedure was a lumenless lead; therefore, there were no results for stylet‐driven leads. Finally, only one study reported clinical outcomes, including HF hospitalization and mortality. Thus, large‐scale randomized controlled trials with long‐term observations are needed.

## 5 CONCLUSION

In all studies included in this meta‐analysis, LBBAP in patients with HFmrEF was feasible and safe. In the pooled analysis, LBBAP was found to significantly shorten the QRS duration and improve systolic cardiac function in patients with an LVEF of 35%−50%. This suggests that the application of LBBAP as a resynchronization strategy for patients with HFmrEF could be an acceptable option, especially in patients with both HFmrEF and dyssynchrony, where a decrease in LVEF is anticipated. A randomized, prospective study is warranted to evaluate the effect of LBBAP‐CRT on patients with HF and an LVEF >35% and ≤35%.

## AUTHOR CONTRIBUTIONS

Ga‐In Yu and Tae‐Hoon Kim contributed to the conception of the study, searched the articles, independently reviewed all identifed articles for eligibility, performed the data analyses, and wrote the manuscript. Yun‐Ho Cho, Jae‐Seok Bae, Jong‐Hwa Ahn, and Jeong Yoon Jang assisted in data acquisition and review literature. Choong Hwan Kwak helped perform the analysis with constructive discussions. All authors reviewed the manuscript.

## CONFLICT OF INTEREST STATEMENT

The authors declare no conflict of interest.

## Supporting information

Supporting information.

## Data Availability

The data underlying this article will be shared on reasonable request to the corresponding author.
