# Supplementary material for: Left bundle branch area pacing in mildly reduced heart failure: A systematic literature review and meta‐analysis
Source: Clin Cardiol. 2023 May 5;46(7):713–20. doi: 10.1002/clc.24028 (PMC10352976; doi:10.1002/clc.24028)
Supplement: Supplementary file 1 — Supporting information. [file CLC-46-713-s001.docx]

**Supplementary Table S1.** The quality of studies included in the final analysis, based on the Newcastle-Ottawa Scale

| Study | Selection | | | | Comparability | Exposure | | | Total (0-9) |
| --- | --- | --- | --- | --- | --- | --- | --- | --- | --- |
|  | Adequate definition of cases | Representativeness of cases | Selection of controls | Definition of controls | Control for important factor or additional factor | Ascertainment of exposure | Same method of ascertainment for cases and controls | Non-response rate |  |
| Li et al, 2020^25^ | 1 | 1 | 1 | 1 | 0 | 1 | 1 | 0 | 6 |
| Li et al, 2020^26^ | 1 | 0 | 0 | 0 | 0 | 1 | 0 | 0 | 2 |
| Qian et al, 2020^27^ | 1 | 1 | 1 | 1 | 0 | 1 | 1 | 0 | 6 |
| Ponnusamy et al, 2021^28^ | 1 | 0 | 0 | 0 | 0 | 1 | 0 | 0 | 2 |
| Wei et al, 2021^29^ | 1 | 0 | 0 | 0 | 0 | 1 | 0 | 0 | 2 |
| Vijayaraman et al, 2021^30^ | 1 | 1 | 1 | 1 | 0 | 1 | 1 | 0 | 6 |
| Jiang et al, 2022^31^ | 1 | 1 | 1 | 1 | 0 | 1 | 1 | 0 | 6 |
| Rademakers et al, 2022^32^ | 1 | 0 | 0 | 0 | 0 | 1 | 0 | 0 | 2 |

**Supplementary Figure S1. Begg’s funnel plots for LBBAP**

**
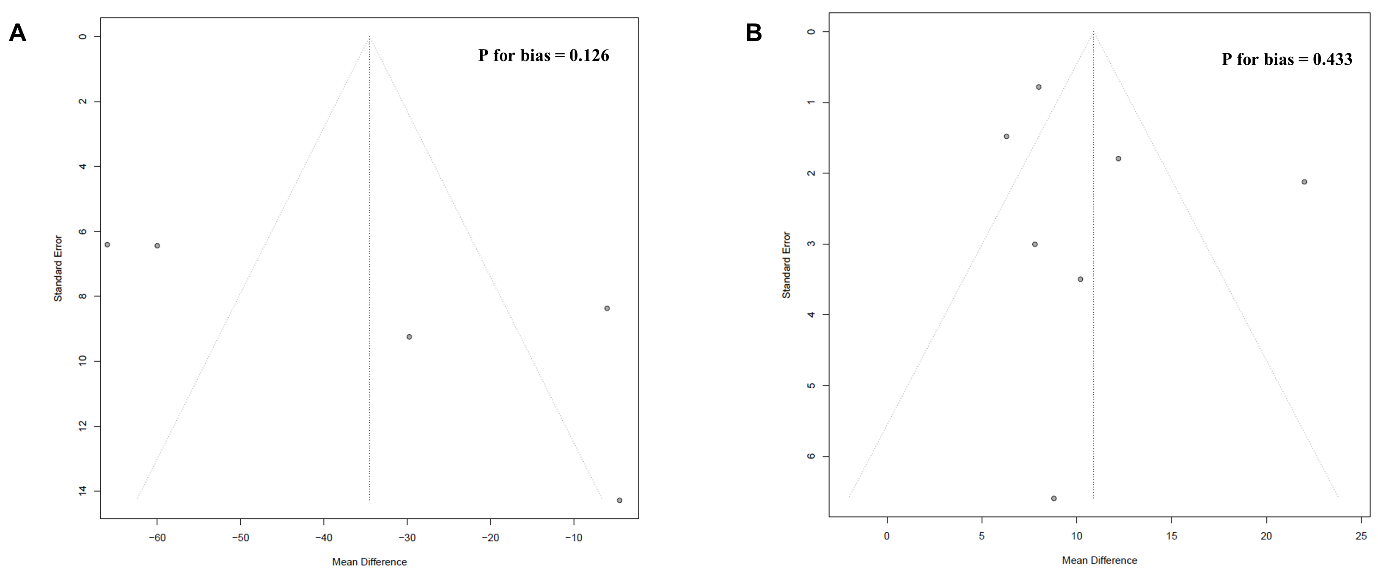
**

A. QRS duration. B. Left ventricular ejection fraction.

The plot demonstrating there was no obvious publication bias.

LBBAP, left bundle branch area pacing.
